# Antibacterial Activities of a New Brominated Diterpene from Borneon *Laurencia* spp

**DOI:** 10.3390/md8061743

**Published:** 2010-05-26

**Authors:** Charles Santhanaraju Vairappan, Takahiro Ishii, Tan Kai Lee, Minoru Suzuki, Zhan Zhaoqi

**Affiliations:** 1 Laboratory of Natural Products Chemistry, Institute for Tropical Biology and Conservation, Universiti Malaysia Sabah, 88999 Kota Kinabalu, Sabah, Malaysia; 2 Shimadzu (Asia Pacific) Pte Ltd, 16 Science Park Drive, #01-01, The Pasteur Singapore Science Park, 118227, Singapore

**Keywords:** *Laurencia* sp., halogenated metabolites, antibacterial activity

## Abstract

In our continuous interest to study the diversity of halogenated metabolites of Malaysian species of the red algal genus *Laurencia*, we examined the chemical composition of five populations of unrecorded *Laurencia* sp. A new brominated diterpene, 10-acetoxyangasiol (**1**), and four other known metabolites, aplysidiol (**2**), cupalaurenol (**3**), 1-methyl-2,3,5-tribromoindole (**4**), and chamigrane epoxide (**5**), were isolated and identified. Isolated metabolites exhibited potent antibacterial activities against clinical bacteria, *Staphylococcus aureus, Staphylococcus* sp., *Streptococcus pyogenes, Salmonella* sp. and *Vibrio cholerae.*

## 1. Introduction

Red algae of the genus *Laurencia* (Rhodomelaceae, Ceramiales) are known to be prolific sources of a wide variety of halogenated secondary metabolites, such as C_15_-acetogenins and C_15_-, C_20_-, and C_30_-terpenoids [1]. In the course of our chemical and biological investigation of *Laurencia* species from the coastal waters of Borneo (Malaysia), we reported the chemical composition of *L. snackeyi* (Weber-van Bosse) Masuda [2], *L. similis* Nam et Saito [3], and *L. majuscula* (Harvey) Lucas [4,5]. As part of the chemical analysis of the undescribed *Laurencia* species, we examined five populations of unrecorded *Laurencia* sp. collected from the coastal waters of North Borneo Island, Sabah. Each specimen contained one halogenated metabolite, a total of five halogenated metabolites were isolated and identified. These specimens yielded one new 10-acetoxyangasiol (**1**) and four known halogenated metabolites; aplysiadiol (**2**), cupalaurenol (**3**), 1-methyl-2,3,5-tribromoindole (**4**), and chamigrane epoxide (**5**). The structure of the new compound 10-acetoxyangasiol (**1**) was elucidated by spectral data. The structures of the known metabolites (**2**–**5**) were determined based on the comparison of spectral data to that of the published reports of Ojika *et al.* [6,7], Ichiba and Higa [8] and Carter and Rinehart [9]. In this paper, we describe the isolation and structure elucidation of these compounds and their antibacterial activities against clinical bacteria.

## 2. Results and Discussion

**Figure 1:**
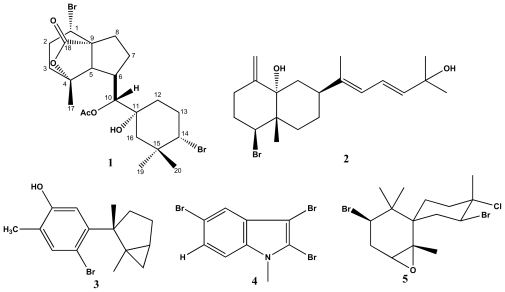
The structure of compounds **1**–**5** from specimens of algae *Laurencia* sp.

The partially dried specimens of algae *Laurencia* sp. were extracted in MeOH (1:1, v/v). The concentrated extracts were partitioned between H_2_O and EtOAc. The EtOAc soluble fraction was dehydrated over Na_2_SO_4_ anhydrous, filtered, concentrated and purified by a combination of silica gel column chromatography and High Performance Liquid Chromatography (HPLC) separation, to yield compounds **1**–**5** (Figure 1).

Compound **1** was obtained as white powder, [α]^25^_D_ +6.4 (*c* 0.9, CHCl_3_). The IR spectrum indicated the presence of OH group (3532 cm^−1^), γ-lactone carbonyl group (1773 cm^−1^) and acetoxy functionality (1734 and 1236 cm^−1^). The positive ESI-MS exhibited a characteristic molecular-ion cluster at *m*/*z* 535/537/539 in a ratio of 1:2:1, suggesting the presence of two bromine atoms. The molecular formula was determined to be C_22_H_32_Br_2_O_5_ by HR-ESI-TOFMS, indicating six degrees of unsaturation. The ^13^C NMR spectrum (Table 1) along with the DEPT experiments showed the presence of 22 carbons including four methyls, seven methylenes, five methines and six quaternary carbon atoms. In addition, the ^1^H and ^13^C NMR spectral data (Table 1) indicated the presence of a γ-lactone carbonyl group [δ_C_ 174.3 (s)] and an associated tertiary alkoxy group [δ_C_ 83.0 (s)], an acetoxy group [δ_C_ 169.4 (s), 20.2 (q); δ_H_ 1.58 (3H, s)], an acetoxymethine [δ_C_ 83.0 (d); δ_H_ 4.31 (1H, d, *J* = 8.9 Hz)], a tertiary alcohol [δ_C_ 73.7 (s)], two bromomethines [δ_C_ 64.4 (d); δ_H_ 3.52 (1H, dd, *J* = 13.1, 4.1 Hz) and δ_C_ 51.0 (d); δ_H_ 3.33 (1H, dd, *J* = 11.7, 6.2 Hz)] and three tertiary methyls [δ_H_ 1.25, 1.15 and 0.93 (each 3H, s)]. According to the molecular formula and the functionalities mentioned above, compound **1** is suggested to contain one γ-lactone and three carbocyclic rings. Furthermore, the ^13^C-NMR spectra of **1** closely resembled those of the known compound angasiol [10], except for the absence of one acetoxy group. It clearly suggested that **1** possesses the same skeleton and substituent.

**Figure 2:**
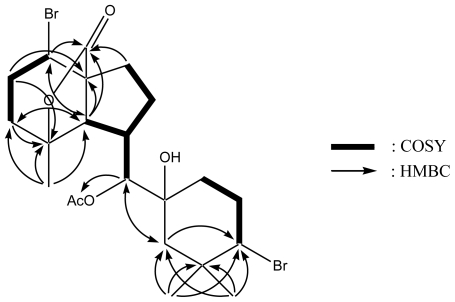
^1^H-^1^H COSY correlations (bold lines) and key HMBC correlations (H→C) of **1**.

**Figure 3:**
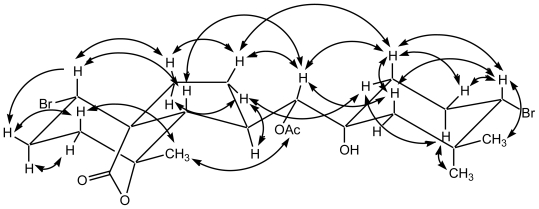
NOESY correlations of **1**.

Assignments were carried out based on ^1^H-^1^H COSY, HSQC and HMBC spectra data. ^1^H-^1^H COSY experiments revealed the sequences of the correlations depicted by the bold lines in Figure 2. Key HMBC correlations as shown in Figure 2 were consistent with the proposed structure of **1**. Furthermore, the relative stereochemistry of **1** was elucidated by NOESY experiments as well as the coupling constants in the ^1^H-NMR spectrum. The coupling constant (*J*_6,10_ = 8.9 Hz) indicated the *anti* conformation for the H-6/H-10. The configuration at C-10 was assigned as R* on the basis of the NOESY correlations observed between H-5/H-10, H-7β/H-10, H-10/Hax-12, H-10/Hax-16 and H_3_-17/H_3_-Ac. In addition, the remaining stereochemistry of **1** was determined by NOESY correlations as shown in Figure 3 and assigned to be the same as for angasiol and its related compounds, irieols A–G [10–12]. In consequence, the structure of 10-acetoxyangasiol (**1**) must be represented by structure **1**.

All five metabolites were subjected to antibacterial bioassay against five species of clinical bacteria, and their antibacterial activities at 30 mg disc^−1^ are shown in Table 2. Compounds **1**, **2** and **3** exhibited potent inhibition against three of the tested bacteria. The lowest MIC value was observed for compound **1** against *Vibrio cholerae* at 100 μg mL^−1^. Compounds **4** and **5** only exhibited weak inhibition against *Staphylococcus* sp. with a MIC value of 300 μg mL^−1^. Vancomycin, used as a positive control, exhibited a >23 mm inhibition zone against all the tested microbes at 30 mg disc^−1^. Based on our findings, these halogenated metabolites could be considered as possible candidates for further investigation against clinical microbes in our endeavor to combat the rise in antibiotic resistant microbes.

## 3. Experimental Section

### 3.1. General

Optical rotations were measured on an AUTOPOL IV automatic polarimeter (Rudolph Research Analytical). ^1^H-NMR (600 MHz) and ^13^C-NMR (150 MHz) spectra were recorded with a JEOL ECA 600, with TMS as internal standard. HR-ESI-TOFMS spectrum was obtained with LCMS-IT-TOF (Shimadzu). Silica gel (Merck, Kieselgel 60, 70–230 mesh) was used for column chromatography. Separation of the eluted fraction were carried out using Shimadzu HPLC with Phenyl Hexyl (Phenomenex, USA) 10 × 250 mm eluted with 70% MeCN/H_2_O, and detected at 210 nm using SPD 20A Shimadzu UV-Vis detector. Analytical TLC was performed on Merck Kieselgel 60 F_254_. Spots were visualized by UV light or by spraying with a 5% phosphomolybdic acid-ethanol solution.

### 3.2. Algal material

Specimens of *Laurencia* sp. were collected from Lohok Butun (4°27’23”N, 118°41’12”E), Selakan Island (4°35’00”N, 118°42’04”E), Layangan Island (5°46’63”N, 115°53’39”E), and Gaya Island (6°00’47”N, 116°03’14”E). The specimens (voucher nos. 37604, 37610, 37665, 37767, 37774) are deposited in the Herbarium (BORH) of Institute for Tropical Biology and Conservation, Universiti Malaysia Sabah.

### 3.3. Extraction and isolation

The partially dried alga specimens (100 g) were extracted with MeOH. The MeOH solution was concentrated *in vacuo* and partitioned between EtOAc and H_2_O. The EtOAc fraction was washed with water, dried over anhydrous Na_2_SO_4_, and evaporated to leave dark green oil. The extract was then fractioned by Si gel column chromatography with a step gradient (hexane and EtOAc). The fractions eluted with hexanes-EtOAc (9:1) and hexanes-EtOAc (8:2) were further purified by HPLC to give 10-acetoxyangasiol (**1**) (2.4%) from specimen BORH 37610, aplysidiol (**2**) (3.2%) from specimen BORH 37665), cupalaurenol (**3**) (0.9%) from specimen BORH 37774, 1-methyl-2,3,5-tribromoindole (**4**) (2.8%) from specimen BORH 37767, and chamigrane epoxide (**5**) (3.6%) from specimen BORH 37774. Yield was calculated based on the extracted methanol crude.

### 3.4. Characterization of 10-acetoxyangasiol (**1**)

White powder; [α]^25^_D_ +6.4 (*c* 0.9, CHCl_3_); HR-TOFMS *m*/*z* 535.0661 [M + H]^+^ (calculated for C_22_H_33_Br_2_O_5_, 535.0689); ^1^H-NMR and ^13^C-NMR spectral data: see Table 1.

### 3.5. Bioassay

The antibacterial bioassay for the isolated metabolite was carried out against 5 species of clinical pathogens obtained from Department of Pathology, Queen Elizabeth Hospital, Kota Kinabalu, Sabah, Malaysia. These bacteria are *Staphylococcus aureus* (UMS01-08)*, Staphylococcus* sp. (UMS02-08), *Streptococcus pyogenes*(UMS03-08)*, Salmonella* sp. (UMS04-08) and *Vibrio cholerae*(UMS05-08). One loopful of each organism was precultured in 20 mL of peptone water overnight. The turbidity of the culture was adjusted to an optical density (OD) of McFarland 0.5 [13]. Then 0.1 mL of the precultured bacterial suspension was used to seed Nutrient Agar plates. Paper discs (Whatman, 6 mm) impregnated with 30 mg disc^−1^ of the respective isolated compounds were placed on the seeded agar plates and the diameter of the inhibitory zones measured after incubation at 28 °C for 24 h. Antibacterial activity was evaluated by measuring the diameter of inhibition zone of the tested bacteria. Vancomycin (Sigma, Germany) was used as positive control. Minimum Inhibitory Concentration (MIC) determination for the positive inhibitions was carried out via microdilution broth method as described by Shan *et al.* (2008) with slight modifications [14].

## 4. Conclusions

This is our first report on the composition of halogenated metabolites and their activities in unrecorded *Laurencia* sp. from the coastal waters of North Borneo Island of Sabah, Malaysia. Our initiative pertaining to the isolation and identification of halogenated secondary metabolites from Borneon *Laurencia* continues to excite us, with the isolation of a wide diversity of structurally interesting metabolites. To date, we have isolated a total of 42 halogenated metabolites from *L. snackeyii*, *L. majuscula*, *L. similis*, *L. nangii* and these five species of undescribed *Laurencia* spp.

## Figures and Tables

**Table 1:** ^1^H-NMR and ^13^C-NMR spectral data of compound **1** (recorded at 600/150 MHz in CDCl_3_; δ in ppm, *J* in Hz).

C	^13^C (δ)	^1^H (δ)	Multiplicity (*J* in Hz)
1	51.0	3.33	(dd, 11.7, 6.2)
2	32.6	2.00	m
		1.82	m
3	38.1	1.32–1.39	m
		0.81	(ddd, 13.7, 12.4, 5.5)
4	83.0		
5	60.1	1.34	(d, 8.3)
6	38.0	2.32	(dddd, 11.0, 8.9, 8.3, 4.8)
7	29.6	1.50–1.57	m
		1.05	(dddd, 14.5, 10.3, 4.8, 2.0)
8	32.7	2.54	(ddd, 13.1, 8.3, 2.0)
		1.43	(ddd, 13.1, 10.3, 10.3)
9	61.8		
10	83.0	4.31	(d, 8.9)
11	73.7		
12	34.3	0.90–0.95	m
		0.56	(ddd, 13.1, 13.1, 4.1)
13	29.7	2.25	(dddd, 13.1, 13.1, 13.1, 3.4)
		1.78	(dddd, 13.1, 4.1, 4.1, 4.1)
14	64.4	3.52	(dd, 13.1, 4.1)
15	36.1		
16	47.2	1.29	(dd, 13.7, 3.4)
		0.92	(d, 13.7)
17	21.9	1.15	s
18	174.3		
19	22.7	1.25	s
20	32.3	0.93	s
Ac	169.4		
	20.2	1.58	s

**Table 2:** Diameter of inhibition zone (DIZ) and minimum inhibitory concentration (MIC) of halogenated metabolites from *Laurencia* sp against five bacteria.

Tested Bacteria	Halogenated Metabolites				
	1	2	3	4	5

***INHIBITION AT 30 mg/disc (mm)***					
*Staphylococcus aureus*	10	8	15	-	-
*Staphylococcus* sp.	12	10	15	9	9
*Streptococcus pyogenes*	-	-	-	-	-
*Salmonella* sp.	-	9	15	-	-
*Vibrio cholerae*	18	-	11	-	-
***MINIMUM INHIBITION CONCENTRATION (MIC) (****μ****g/mL)***					
*Staphylococcus aureus*	250	200	125	-	-
*Staphylococcus* sp.	200	125	125	300	300
*Streptococcus pyogenes*	-	-	-	-	-
*Salmonella* sp.	-	250	125	-	-
*Vibrio cholerae*	100	-	200	-	-

Note: Bioassay were done in triplicate, SD <10%, and not shown.
